# Laparoscopic Retrograde Nephrectomy as a Troubleshooting Technique to Prevent Open Conversion: The Technique Description With a Review of 40 Cases

**DOI:** 10.7759/cureus.61482

**Published:** 2024-06-01

**Authors:** Deerush Kannan, Nandyala Penchala Reddy, Aarthy Paneerselvam, Rajesh Paul, Mathisekaran Thangarasu, Vinayak Rengan, Nitesh Jain

**Affiliations:** 1 Urology, Apollo Hospitals, Chennai, IND; 2 Surgery, Apollo Hospitals, Chennai, IND

**Keywords:** genitourinary tuberculosis (gutb), renal tumour, difficult nephrectomy, laparoscopic radical nephrectomy, open nephrectomy

## Abstract

Introduction

Laparoscopic nephrectomies are safe, with low complication rates in skilled hands. However, traditional approaches may be unsuitable for conditions such as post-renal abscesses, long-standing urinomas, non-functioning kidneys post-pyeloplasty, pyelolithotomies, post-partial nephrectomy recurrences, tuberculous kidneys, pyelonephritis, and redo-renal surgeries. This study describes a modified retrograde nephrectomy technique and its outcomes in 40 cases.

Methods

We reviewed 40 cases where the retrograde nephrectomy technique was used. Surgeons opted for this method based on intraoperative findings and initial difficulties in accessing the lower pole area.

Results

Traditional dissection was challenging due to adhesions in the lower pole. The retrograde technique, starting from the renal hilum, allowed early ligation of renal arteries and veins, reducing bleeding risks and facilitating safer caudal dissection.

Conclusions

The retrograde nephrectomy technique offers a safer and more efficient alternative for complex nephrectomies. Early vascular control minimizes hemorrhage risk, making it a valuable method in challenging renal surgeries.

## Introduction

Laparoscopic nephrectomies are performed by urologists frequently for a variety of reasons. The procedure is well-established and follows the same process as an open surgery [1]. The standard procedure involves mobilizing the lower pole of the kidney first, followed by dissection to trace the hilum, and isolating and ligating the renal artery and the renal vein. Once hilar control has been obtained, the kidney is mobilized from its cranial and lateral aspects [2]. The most crucial part of the surgery is the management of renal hilar vessels [3]. It requires identifying and meticulously dissecting the renal vessels and their ligation [4]. This needs extensive experience and is the most demanding step for surgeons in the early stages of their careers [5]. Injury to these vessels can result in open conversion and potentially fatal outcomes.

We present a simplified approach for difficult laparoscopic nephrectomy as a bail-out procedure where dissecting in a conventional way can be difficult - a "retrograde procedure" in which the dissection begins at the upper pole of the kidney and progresses to hilar dissection to acquire control of the hilar vessels. In this method, the surgeon completely mobilizes the kidney at a higher level, which is usually a virgin area in this condition, before dealing with the renal hilum. Though this may not be the ideal approach for all nephrectomies, we propose this method as a troubleshooting option in cases where the lower pole area has more adhesions in particular circumstances, such as post-pyeloplasty, post-renal abscess simple nephrectomies, post-lower pole partial nephrectomies, tubercular kidney, long-standing urinoma, where dissection from the lower pole is difficult. In most of these situations, lifting the kidney from the lower pole is more complicated than in regular cases because the infection is housed in the pelvis or ureter, which causes adhesions. Lifting the kidney to access the hilum becomes challenging in patients with a large lower pole renal tumor as well. Approaching the hilum from the upper pole is easy in these cases because the planes are virgin, and once hilar control is obtained, the lower pole dissection can be completed without much bleeding. The procedure is suitable for both simple and radical laparoscopic nephrectomies.

## Materials and methods

This study was performed at our center in the Department of Urology from March 2013 to March 2023. The study adhered to the guidelines outlined in the Declaration of Helsinki and received approval from the Institutional Ethics Committee (IRB number: AMH-C-S-072/09-23). The case records of 40 patients, in whom the retrograde nephrectomy technique was used, were selected for the study. The decision to proceed with this technique was made by the operating surgeon, taking into consideration the intraoperative findings and the difficulty noticed at the time of initial access to the lower pole area. Written consent was obtained from all patients as part of the preoperative process for any surgery.

All procedures were performed by the same surgeon. We extracted data, including demographic information such as age, gender, and side of nephrectomy; patients' preoperative imaging findings and reasons for nephrectomy; intraoperative parameters such as operative time, blood loss, number of renal arteries, and open conversion rate; and postoperative factors such as hospital stay, histopathology findings, and secondary bleeding. The 30-day readmission rate and delayed complications pertaining to the procedure were also extracted.

Instruments needed

A 30-degree telescope; 5 mm and 10 mm ports; laparoscopic tools such as an atraumatic bowel grasper; a self-retaining toothed grasper for hepatic retraction; right-angle Maryland forceps; a Hem-o-lok clip applicator (Teleflex, Wayne, USA); a metallic clip applicator; laparoscopic scissors; and a suction cannula. Depending on availability or comfort, the surgeon can use a monopolar device or Harmonic (Ethicon, Inc., Johnson & Johnson, Somerville, USA) for dissection. Open instruments must always be accessible in the operating room at all times, with the safety of patients being the first concern.

Positioning of the patient

In the supine position, a Foley catheter is inserted, after which the patient is turned to a lateral decubitus position, angled 90 degrees relative to the table. The patient should be positioned close to the table's edge to ensure the surgeon has ample room to move. To access the hilum effectively, a table break is made between the iliac crest and the 12th rib. Attached bolsters and backrests on the table provide ample support to the patient's back. The upper leg is kept straight, while the lower leg is bent at the knee. The arms are flexed and placed on armrests, positioned cranially to avoid obstructing instrument maneuverability. This also allows the anesthesiologist to maintain immediate vascular access. All pressure points are adequately padded to prevent injury.

Creation of pneumoperitoneum

The pneumoperitoneum can be created using either the open or closed method. The general principles of the hanging drop test and saline aspiration test have to be followed. A pneumoperitoneum of 12 mm of mercury has to be achieved.

Port placement for left-side nephrectomy

**Figure 1:**
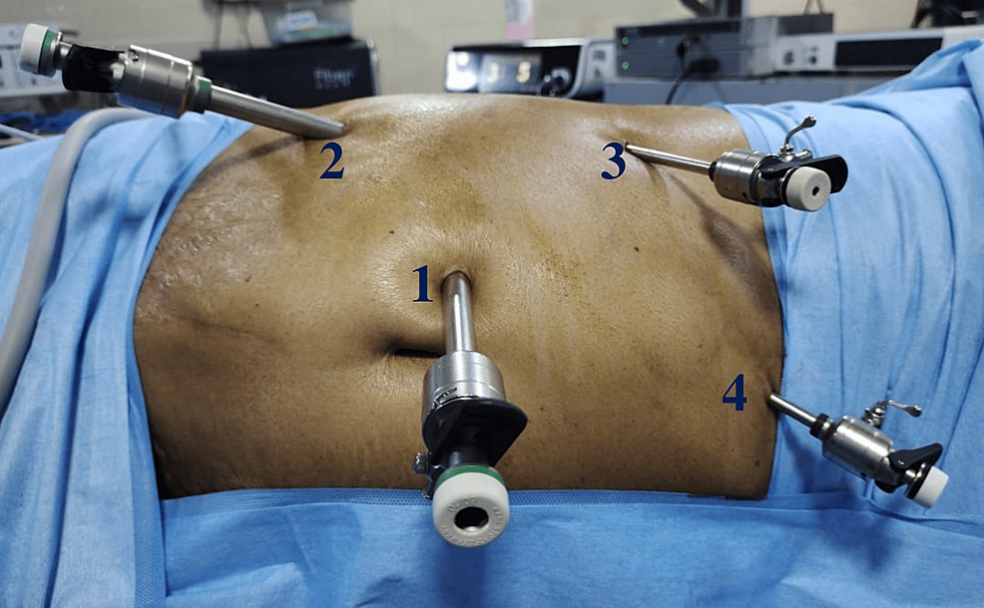
Port placement by triangulation technique 1: camera port; 2 and 3: working ports; 4: liver retraction port

The initial port, a 10-mm port, is positioned slightly lateral to the midpoint of the spinoumbilical line. A 5-mm port is then inserted parallel to the 10-mm port in the sub-costal area. Another 10-mm port, designated as the camera port, is placed using the triangulation method (Figure 1). Finally, a 5-mm port is positioned along the midline in the suprapubic region to help the assistant with intestinal retraction and the suctioning of blood and fumes.

Port placement for right-side nephrectomy

On the right side, the same principles for port placement are followed, but a 5-mm hepatic retraction port is inserted in the epigastric area.

Steps of surgery

Dissection is started in a conventional way. When difficulty is encountered, the following approach is adopted. 

Upper Pole Mobilization

**Figure 2:**
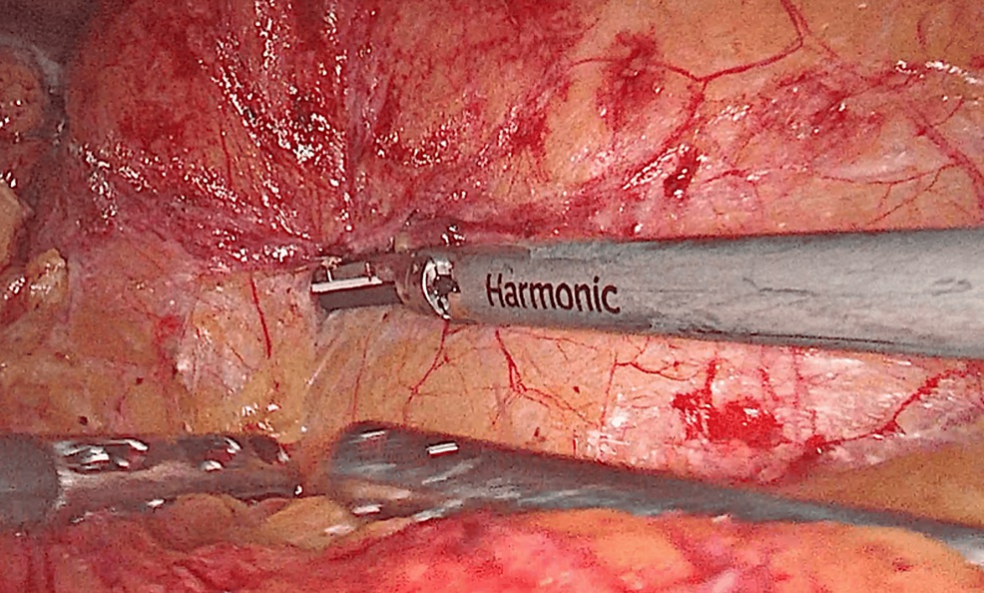
Mobilization of the colon along the avascular line of Toldt

**Figure 3:**
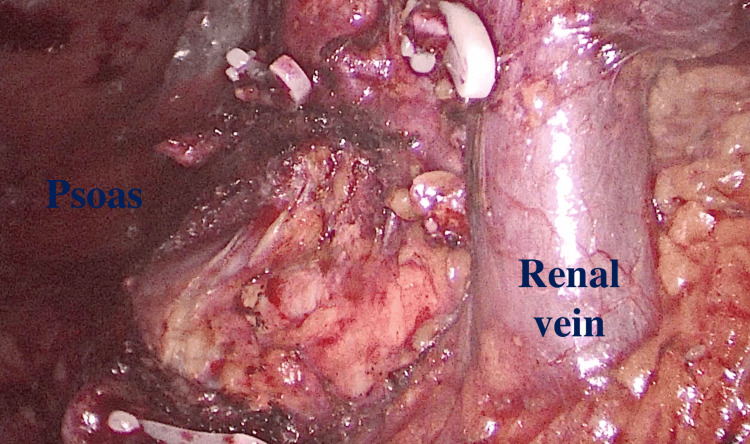
The renal vein is exposed after completing the upper pole dissection The psoas can be appreciated in the upper pole region at this point

The first step is a diagnostic laparoscopy after which the dissection begins with the upper pole. The colon is mobilized for a small region at the level of splenic (left nephrectomy) or hepatic flexure (right nephrectomy). The spleen is mobilized by splitting the splenorenal and splenic-diaphragmatic ligaments and allowing them to descend medially (Figure 2). The triangular ligament on the right side is separated to elevate the liver from the upper pole. At this stage, the aim is to visualize the psoas muscle and diaphragm behind the upper pole (Figure 3). The aim is to remove all adhesions around the upper pole, detaching them from the psoas muscle. Dissection should proceed medially until the renal vein is clearly seen (Figure 3). If the adrenal gland is to be removed, the adrenal vein should be clipped and divided. Any confirmed accessory vessel leading to the upper pole can also be clipped and divided.

Hilar Dissection

After reaching the hilum while preserving the vein, a posterior dissection is carried out to locate the renal artery. The fibrofatty tissues surrounding the renal artery are dissected, either with an energy device or a right angle, so that clips can be applied to occlude the whole lumen of the vessel. The renal artery is double-clipped and divided. At this time, the surgeon can determine if there are any other accessory arteries by occluding the renal vein through a soft device and checking for retrograde filling of the vein. The renal vein is double-clipped and divided after confirmation.

Colon Mobilization

**Figure 4:**
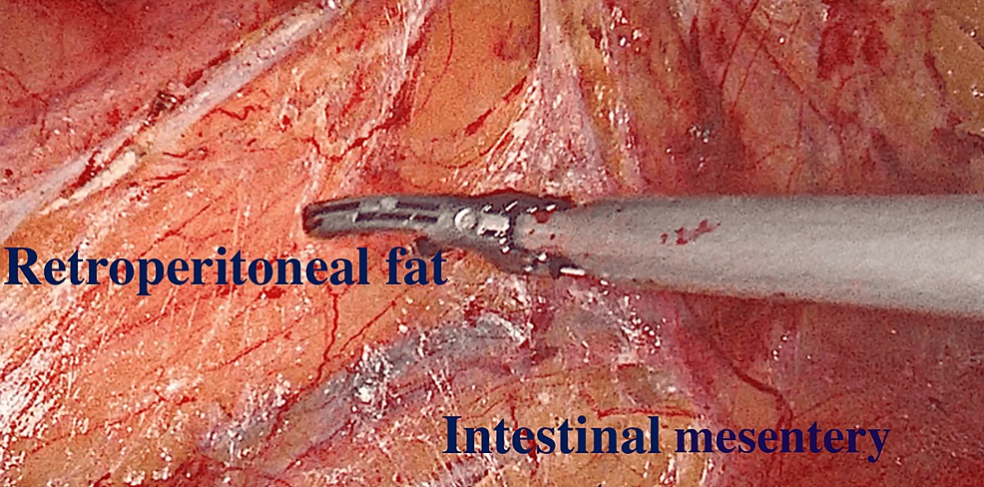
The retroperitoneal fat is dark yellow, whereas the intestinal mesentery appears bright yellow

The incision of the white line of Toldt mobilizes the colon. It is recommended to start at the level of the pelvic brim on the left side and move towards the spleen; for right-sided nephrectomies, starting at the level of the liver on the right side and moving caudally is more ergonomic. The colon has to be completely mobilized to connect with the previously incised section at the upper pole. When employing energy devices at this stage, the surgeon must use extreme caution to prevent unintentional electric or thermal harm to the colon. The retroperitoneal fat has a dark yellow color, but the intestinal mesentery appears bright yellow (Figure 4). This can aid in correctly identifying the plane that connects the mesentery to the retroperitoneum. If there is excessive bleeding, the plane probably needs to be revised, as the bleeding is likely to be from mesenteric veins. Due to adhesions, bleeding is likely at this point, although it may be safely cauterized. At this stage, a blunt tool like suction is quite helpful to sweep the mesentery away. 

Identification of Ureter and Gonadal Vessel

**Figure 5:**
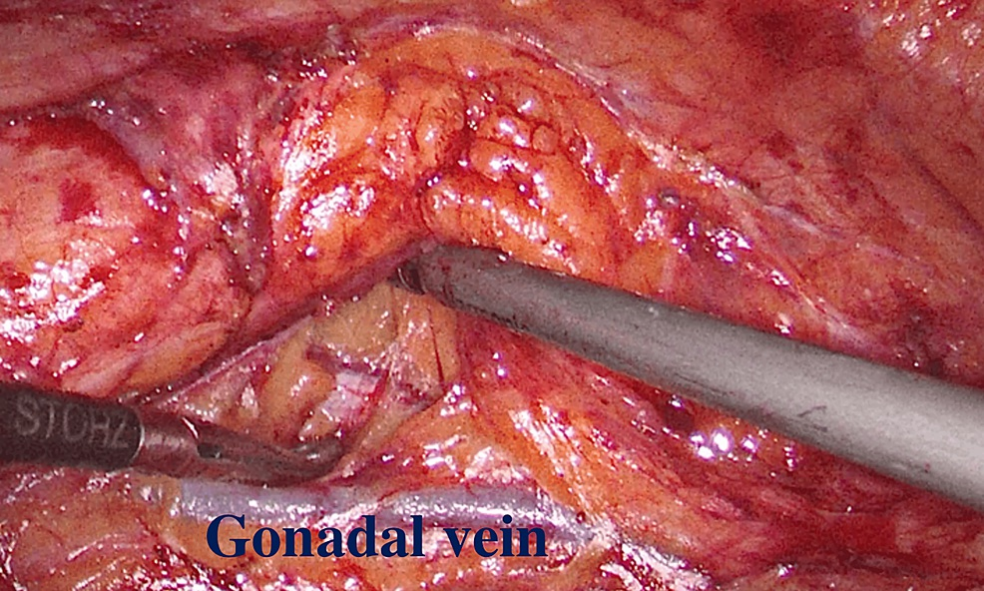
The gonadal vein is being dropped from the area of dissection

The next step is finding the psoas muscle. Now, the surgeon should try to separate the ureter and the gonadal vessels. By cutting through the fat above the gonadal vein on the left side, we drop the gonadal vessels (Figure 5). By doing so, the lumbar veins may be avoided as the dissection moves cranially. We medialize the gonadal vessels on the right side and identify the ureter. 

Lower Pole Mobilization

The fatty tissue between the ureter and the psoas muscle is dissected using both blunt and sharp dissection. Minor ureteric vessels might be present at this point that can be separated with the aid of energy tools. The operating surgeon regularly adjusts the traction on the ureter as he moves closer to the lower pole of the kidney. To give traction for further dissection, the assistant aids in retracting the reflected bowel. The gonadal artery is located directly below the lower pole and can be sacrificed if required. As the hilar control has already been obtained, the lower pole is raised off the psoas, and the kidney is then freed from its attachment on the other sides like in a conventional nephrectomy. If lower-pole accessory vessels are found, they can be controlled at this point. Additionally, the lumbar veins can be observed emptying into the renal vein, particularly on the left side. They should not be touched. After verifying hemostasis, the kidney is bagged, and the specimen is then collected by stretching the port in the lower abdomen.

## Results

There were 42 cases attempted by this technique based on intraoperative findings, out of which there was conversion into open surgery in two of the cases. In both these cases, it was a large renal mass in the lower pole that could not be lifted off the psoas due to dense adhesions (one in xanthogranulomatous pyelonephritis and the other in a patient with locally advanced malignancy who underwent palliative nephrectomy). However both these cases were managed successfully by open surgery. Among the 40 studied cases, there were two cases that had complications. One patient had a small tear on the inferior vena cava (IVC) that required suturing without conversion to open, and the other patient had a bowel perforation that was recognized intra-operatively and was successfully repaired by the same incision at the time of specimen retrieval. Both these patients underwent a cytoreductive nephrectomy. There were no post-operative complications or need for any blood transfusion. The mean hospital stay was 2.4 days. The demographics and perioperative findings are summarized in Table 1 and the final histopathological diagnosis is summarized in Table 2.

**Table 1:** Demographics and perioperative findings

Parameter	Value
Patient total (n)	40
Mean age (years)	44.5
Sex (n)	
Male	24
Female	16
Side of operation	
Left	26
Right	14
Mean preoperative creatinine (mg/dL)	0.9
Mean operative time (mins)	140
Estimated mean blood loss (mL)	184
Blood transfusion (n)	0
Intraoperative complications (n)	2
Mean hospital stay (days)	2.4
Mean postoperative creatinine (mg/dL)	1.1

**Table 2:** Final histopathological diagnosis

Diagnosis	Number of cases (n)
Tuberculous kidney	4
Renal malignancy	6
Large lower pole mass	4
Completion radical nephrectomy	2
Non-functioning kidney	24
Stone disease causing pyelonephritis	18
Pelviureteric junction obstruction	6
Nephroureterectomy with pelvic tumor	3
Xanthogranulomatous pyelonephritis	3

## Discussion

In the early 1990s, the very first laparoscopy nephrectomy was done, and it has since evolved into the accepted standard of care for treating kidney diseases [6]. Smaller incisions, less discomfort, quicker healing, and shorter hospital stays are the key benefits of laparoscopic nephrectomy versus open surgery [7]. The time required for surgery and complications has dramatically reduced because of technical developments, surgical improvement, greater energy accessibility, and ultrasonic instruments.

While nephrectomy is often the initial minimally invasive surgery taught to urology trainees, it requires a significant learning curve. To get through this learning curve, it has been discovered that 20 to 50 cases or a minimum of 12 months of focused training at a tertiary care center may be required [8]. The treatment of renal hilum is the major problem in the procedure. The kidneys are very vascular organs that receive a considerable portion of the total cardiac output. Additionally, the aorta and the IVC are directly connected to the renal arteries and veins, respectively. The location of the renal artery beneath the vein adds complexity to identification, especially for trainees [9]. Most difficulties during the learning curve's early stages occur during the intra-hilar dissection of the arteries and veins. This included vena cava injuries as well as damage to the primary renal vein [10]. 

Where it’s a well-known fact that most catastrophes occur at this juncture, having a bail-out method like the retrograde technique is always a better option prior to open conversion in the discussed indications [11]. Though there were two complications noted in this study, it cannot be assured that the same could have been avoided if the procedure was converted into open; this could be attributed to the pre-existing difficulty in distorted anatomy rather than the technique. This could potentially reduce the rate of open conversion in re-operative cases at the same time without compromising patient safety. This can be an easily adaptable troubleshooting technique like the two window and en-bloc techniques [12,13].

This study is not without limitations. Being a retrospective study, it is prone to bias. When it comes to the technique, it is to be noted that if there are any lower pole vessels, they could be a potential source of bleeding when the dissection is started from the upper pole. The close proximity of the lumbar vessels around the lower pole could still be a cause of concern in some patients with intense adhesions in the lower pole. Since the primary surgeon has a track record of more than 1000 laparoscopic nephrectomies, the complication rates might be underestimated. Therefore, further prospective studies in a relatively mixed population of surgeons could validate this technique. 

## Conclusions

Retrograde nephrectomy by laparoscopic approach is a valuable technique in the surgical arsenal, with the potential to reduce the need for open conversion in carefully selected cases. Even if open conversion becomes necessary in an elective situation, this technique can serve as a last resort before resorting to an open incision.
